# Heat Transport during Colloidal Mixture of Water with Al_2_O_3_-SiO_2_ Nanoparticles within Porous Medium: Semi-Analytical Solutions

**DOI:** 10.3390/nano12203688

**Published:** 2022-10-20

**Authors:** Hashim, Muhammad Hafeez, Nidhal Ben Khedher, Sayed Mohamed Tag-ElDin, Mowffaq Oreijah

**Affiliations:** 1 Department of Mathematics & Statistics, The University of Haripur, Haripur 22620, Pakistan; 2 Department of Mathematics & Statistics, Riphah International University, Islamabad 44000, Pakistan; 3 Department of Mechanical Engineering, College of Engineering, University of Ha’il, Ha’il 81451, Saudi Arabia; 4 Laboratory of Thermal and Energy Systems Studies, National School of Engineering of Monastir, University of Monastir, Monastir 5000, Tunisia; 5 Faculty of Engineering and Technology, Future University in Egypt, New Cairo 11835, Egypt; 6 Mechanical Engineering Department, College of Engineering and Islamic Architecture, Umm Al-Qura University, Makkah 21955, Saudi Arabia

**Keywords:** hybrid nanofluid, stretching/shrinking sheet, dual solutions, semi-analytical solution, porous medium

## Abstract

In recent years, energy consumption has become an essential aspect in the manufacturing industry, and low heat transfer is one of the obstacles that affect the quality of the final product. This situation can be managed by suspending nanoparticles into ordinary heat transferring fluid (the base fluid). This newly prepared colloidal suspension has better heat transport capabilities. Keeping such usage of nanofluids in mind, this research was performed to better understand the heat transport characteristics during flow analysis saturated in porous media subject to Al2O3-SiO2/water hybrid nanofluids. This flow problem was generated by a stretching/shrinking surface. The surface of the sheet was under the influence of mass suction and second-order partial slip. The boundary layer flow was formulated in a system of partial differential equations by utilizing basic conservation laws in conjunction with the Tiwari and Das nanofluid model. Then, the appropriate form of the similarity transformation was adapted to transform the model into a system of ordinary differential equations. The built-in function, i.e., the bvp4c function in the MATLAB software, solved the reduced form of the boundary layer model. The novelty of this study lay in the predicting of two different exact and numerical solutions for both the flow and temperature fields. The computed results showed that the medium porosity as well as the nanoparticle volume fraction widened the existence range of the dual solutions. In addition, the investigational output exposed the fact that the temperature fields were significantly enhanced by the higher nanoparticle volume fraction. Moreover, the outcomes of this study showed a superb correlation with existing works. The present results can be utilized in various branches of science and engineering such as the polymer industry and in the treatment of different diseases.

## 1. Introduction

Energy loss due to heat transport and friction has severely hampered industrial progress in this era of advancement in science and technology. Efficient heat transfer to heating components and the enhancement of the abrasion resistance of long-term moving components is essential for technological growth. A new procreation fluid with excellent thermal performance would be beneficial for fulfilling the industrial or technological needs to deal with this problem. Choi and Eastman [1] invented a whole new heat transfer fluid based on nanotechnology known as a nanofluid. Nanofluids are indeed produced by combining a base fluid (i.e., water, ethylene glycol) with a mixture of nanoparticles (i.e., metal oxides, metals, carbon materials). Several recent studies have shown both experimentally and theoretically that nanoparticles have outstanding potential to improve the rate of heat transfer of ordinary base fluids and have excellent thermophysical performance. This special class of heat transfer fluids, i.e., nanofluids, can be applied in many fields such as pharmaceutical processes, aerospace, transportation, automotive, air-conditioning, electronics, and power generation. A great deal of work has been reported on the behavior and characteristics of nanofluids in numerous problems since Choi first introduced nanofluids. In this regard, various mathematical models for nanofluids have been reported by various researchers. This includes the two-phase model given by Buongiorno [2] and the single-phase model proposed by Tiwari and Das [3]. Later, a smart transformation was conducted on nanofluids with the aim of enhancing the productivity of the fluids. This is known as hybrid nanofluid, where more than one nanoparticle is mixed in one base fluid. Hybrid nanofluids also have a huge contribution in heat exchangers, machining, and the manufacturing of coolants and heat pipes. Owing to the various potential applications of hybrid nanofluids, various researchers have carried out their research on hybrid nanofluids with inclusion of different physical aspects related to flow and heat transfer mechanism. In this regard, Devi and Devi [4] exhibited a comparative study to reveal the heat transport features of nanofluids and emerging hybrid nanofluids. They presented a new model for the thermophysical properties of hybrid nanofluids during three-dimensional flow over a stretching surface. Again, Devi and Devi [5] investigated the heat transport characteristics of a Cu/Al_2_O_3_-water hybrid nanomaterial. Yousefi et al. [6] then envisaged the heat transfer of a titania-copper hybrid nanofluid towards a wavy cylinder in the vicinity of a stagnation point. Hayat et al. [7] bestowed a numerical study to compare the heat transfer improvement for Ag/CuO-water hybrid nanofluids with the inclusion of partial slip and a rotating system. Later, Usman et al. [8] reported a three-dimensional free-convection rotating flow of Cu-Al_2_O_3_/H_2_O hybrid nanofluids past a permeable stretching surface with thermal radiation impacts. Due to the increasing usage of hybrid nanofluids in real-world settings, researchers have examined theoretical models of hybrid nanofluids by incorporating different physical mechanisms, which can be seen in the works in [9,10,11,12,13,14].

Keeping in view the recent developments in science and technology, we are always seeking for some techniques that produce quality products in a short time. To meet these standards, science and technology have made outstanding progress in several fields. Fluid mechanics is one of the sciences in which researchers and scientists have made excellent progress. In the recent past, several researchers have studied fluid flow owing to a stretching sheet because of its realistic applications in the engineering industry. This can be applied to the manufacturing of paper and glass, the drawing of plastic films, fiber spinning, crystal growing, polymer extrusion etc. To explore the various engineering aspects, Sakiadis [15] started a boundary layer flow past a solid surface. After his pioneering work, Crane [16] extended this by focusing on the distinctive features of stretching surfaces. He was the pioneer who deemed similar solutions for flow driven by a stretching flat plate and obtained an exact analytical solution. Later, Gupta and Gupta [17] explored the heat transport mechanisms during flow past a stretching flat plate under suction and blowing. Later, Wang [18] explored the three-dimensional flow of a Newtonian viscous fluid driven by a flat plate. He produced closed-form analytical solutions. Subsequently, several researchers have developed various physical models to study the distinct aspects of stretching sheet phenomena, which are shown in the references [19,20,21].

On the other hand, numerous researchers have conducted their studies on flow over a shrinking surface in recent years. This new kind of flow driven by a shrinking surface is a backward flow and has adverse features as compared to stretching flow. Goldstein initially pondered this [22] and suggested that mass suction is required to maintain the flow. In other work, Miklavcic and Wang [23] explored the steady flow of a viscous fluid past a shrinking sheet. They obtained the exact solutions of the Navier–Stokes equations and revealed that the flow could be maintain over a shrinking surface by the imposition of mass suction at the boundary. Fang and Zhang [24] investigated the heat transport features during flow over a shrinking sheet. They computed the closed-form solution in the case of some special parameters. The magnetohydrodynamic unsteady flow of viscous fluid driven by a shrinking surface was reported by Merkin and Kumaran [25]. Fang, et al. [26] presented viscous flow driven by a shrinking sheet in the presence of second-order slip mechanism. Apart from above mentioned works, several studies have been reported by various authors concerning boundary layer flow over a shrinking sheet, as shown in the references [27,28,29].

In the recent past, a few analysts have focused on flow induced by a shrinking surface to compute multiple numerical solutions with the help of different available commercial software. These solutions are more important because they allow the depiction of a physically realistic solution for the considered problems. A few studies have been reported in current years that obtained multiple-nature closed-form solutions of an exponential type for fluid flow over a shrinking surface. Andersson [30] obtained multiple exact solutions for viscous fluid flow with partial slip conditions. Later, Wang [31] presented the flow of a Newtonian fluid near a stagnation point. He found the exact closed-form solutions for their problem. Again, Wang [32] revealed the exactly similar solutions for the two-dimensional flow of a viscous fluid generated by a stretching sheet. Furthermore, reviews on the exact multiple solutions for flow and heat transport have been conducted by Fang and Aziz [33], Turkyilmazoglu [34], Aly and Vajravelu [35], Nandy and Pop [36], and Roşca, et al. [37].

### Novelty of This Study

After a careful review of the literature mentioned above, we concluded that various works have been reported on the nanofluid and hybrid nanofluid flow and heat transport problems under different physical situations. However, heat transport optimization during the slip-flow of Al_2_O_3_-SiO_2_/water hybrid nanofluids saturated in porous media has not been considered yet. Additionally, modern technology advancements have increased the interest in improved thermal processes. Utilizing nanoparticles has made it possible to meet growing demand by a substantial amount. Therefore, the primary focus of this study was to try filling this knowledge gap. As a novelty, exact analytical solutions for flow distributions and numerical solutions for thermal distributions were computed for Al_2_O_3_-SiO_2_/water hybrid nanofluids subject to the Tiwari and Das model and the slip mechanism. A two-dimensional mathematical model using Cartesian coordinates was formulated for the incompressible flow of viscous hybrid nanofluids saturated in porous media. As a result, the goal of this study was to determine the effects of critical parameters such as nanoparticle volume fraction, mass suction parameter, first- and second-order slip parameters, the stretching/shrinking parameter, and porosity parameters. An overall correlation of the velocity, temperature, skin-friction coefficient, and solution domain with all the critical flow parameters were plotted graphically. Consequently, the computed results provided a clear picture of how the flow variables affected the behavior of flow and heat transmission.

## 2. Problem Description and Governing Equations

**Figure 1:**
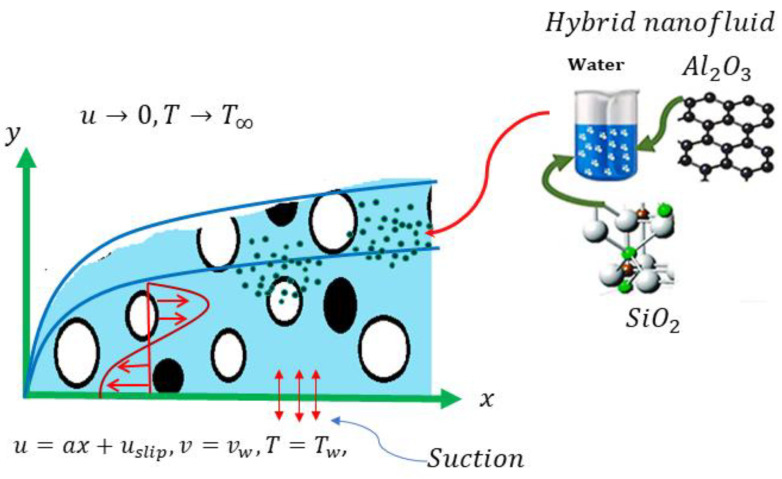
The present problem configuration.

The current work was contemplated with the steady, two dimensional, incompressible, and laminar flow and heat transport features of a hybrid nanofluid (Al_2_O_3_-SiO_2_) generated by a stretching/shrinking surface within a porous media. The Cartesian coordinates ware chosen in such a way that the x−axis extends along the sheet and the y−axis is normal to the surface, being positive in the direction of the surface. The considered physical problem and flow configuration are displayed in Figure 1. The stretching/shrinking sheet has a velocity $U_{w}=ax,$, where a represents a constant while the mass transfer velocity was taken to be $v_{w}.$ In addition, the application of the second-order velocity slip along with porous media was further assimilated in the flow and heat transfer analysis. We assumed that the surface temperature $T_{w}$ and the ambient temperature $T_{\infty }$ are both constant.

In view of above-stated constraints, the governing equations for hybrid nanofluids can be described in the form of continuity, momentum, and energy equations (modified from Aladdin and Bachok [38]):

(1) $$\left(\frac{\partial u}{\partial x}+\frac{\partial v}{\partial y}\right)=0,$$

(2) $$u\frac{\partial u}{\partial x}+v\frac{\partial u}{\partial y}=\frac{\mu_{hnf}}{\rho_{hnf}}\frac{\partial^{2}u}{\partial y^{2}}-\frac{\mu_{hnf}}{\kappa \rho_{hnf}}u-\frac{\sigma_{hnf}}{\rho_{hnf}}B_{0}^{2}u,$$

(3) $$u\frac{\partial T}{\partial x}+v\frac{\partial T}{\partial y}=\frac{k_{hnf}}{{\left(\rho c_{p}\right)}_{hnf}}\frac{\partial^{2}T}{\partial y^{2}}.$$

In the above expressions, u and v the hybrid nanofluid velocities in x and y directions, respectively, T and $k_{o}$ are the hybrid nanofluid temperature and the permeability of the porous media, respectively. The physical aspects of hybrid nanofluids are elucidated in Table 1, $\rho_{hnf},\mu_{hnf},k_{hnf}$ and ${\left(C_{p}\right)}_{hnf}$ namely the density, dynamic viscosity, heat capacity, and thermal conductivity of the hybrid nanofluids. These thermo-physical properties of a hybrid nanofluid are expressed as follows [39,40,41]:

(4) $$A_{1}=\frac{\mu_{hnf}}{\mu_{f}}={\left(1-\varphi_{1}-\varphi_{2}\right)}^{-2.5},$$

(5) $$A_{2}=\frac{\rho_{hnf}}{\rho_{f}}=1-\varphi_{1}-\varphi_{2}+\frac{\varphi_{1}\rho_{s1}+\varphi_{2}\rho_{s2}}{\rho_{f}},$$

(6) $$A_{3}=\frac{{\left(\rho c_{p}\right)}_{hnf}}{{\left(\rho c_{p}\right)}_{f}}=1-\varphi_{1}-\varphi_{2}+\frac{\varphi_{1}{\left(\rho c_{p}\right)}_{s1}+\varphi_{2}{\left(\rho c_{p}\right)}_{s2}}{{\left(\rho c_{p}\right)}_{f}},$$

(7) $$A_{4}=\frac{k_{hnf}}{k_{f}}=\frac{\left[\frac{k_{s1}\varphi_{1}+k_{s2}\varphi_{2}}{\varphi_{1}+\varphi_{2}}+2k_{f}+\left(k_{s1}\varphi_{1}+k_{s2}\varphi_{2}\right)-2\left(\varphi_{1}+\varphi_{2}\right)\;k_{f}\right]}{\left[\frac{k_{s1}\varphi_{1}+k_{s2}\varphi_{2}}{\varphi_{1}+\varphi_{2}}+2k_{f}-\left(k_{s1}\varphi_{1}+k_{s2}\varphi_{2}\right)+\left(\varphi_{1}+\varphi_{2}\right)\;k_{f}\right]},$$

(8) $$A_{5}=\frac{\sigma_{hnf}}{\sigma_{f}}=\left[\frac{\sigma_{2}\left(1+2\varphi_{2}\right)+2\left[\frac{\sigma_{1}\left(1+2\varphi_{1}\right)+2\sigma_{f}\left(1-\varphi 1\right)}{\sigma_{1}\left(1-\varphi_{1}\right)+\sigma_{f}\left(2+\varphi_{1}\right)}\right]\;\left(1-\varphi_{2}\right)\;\sigma_{f}}{\sigma_{2}\left(1-\varphi_{2}\right)+\left[\frac{\sigma_{1}\left(1+2\varphi_{1}\right)+2\sigma_{f}\left(1-\varphi 1\right)}{\sigma_{1}\left(1-\varphi_{1}\right)+\sigma_{f}\left(2+\varphi_{1}\right)}\right]\;\left(2+\varphi_{2}\right)\;\sigma_{f}}\right]\;\left[\frac{\sigma_{1}\left(1+2\varphi_{1}\right)+2\sigma_{f}\left(1-\varphi 1\right)}{\sigma_{1}\left(1-\varphi_{1}\right)+\sigma_{f}\left(2+\varphi_{1}\right)}\right],$$

where, $\rho_{f},$ $\mu_{f},$ ${\left(c_{p}\right)}_{f},$ $k_{f},$ and $\sigma_{f}$ denote the density, viscosity, heat capacitance, thermal conductivity, and electric conductivity, respectively, of the base fluid. However, $\varphi_{1}$, $\varphi_{2}$, $\rho_{s1}$, $\rho_{s2}$, $k_{s1}$, $k_{s2}$, $\sigma_{s1}$, and $\sigma_{s2}$ denote the volume fraction of the nanoparticles, density, thermal conductivity, and electric conductivity of the nanoparticles, respectively. It is important to mentioned here that the subscripts $s_{1}$ and $s_{2}$ refer to the two nanoparticles. The numerical values of the water $-Al_{2}O_{3}$/$SiO_{2}$ hybrid nanofluid employed in this analysis are displayed in Table 1.

The suitable boundary conditions for this problem are [27]:

(9) $$\begin{array}{l} \;\;u=\lambda u_{w}+u_{sip},\;v=v_{w}\left(x\right)\;,\;T=T_{w}\;\mathrm{at}\;y=0,\;\\ u\to 0,\;\;T\to T_{\infty }\;\mathrm{as}\;y\to \infty , \end{array}$$

Here, the slip velocity at the shrinking sheet surface is expressed as $u_{sip}.$ The following slip velocity is valid for an arbitrary Knudsen number, $K_{n}$, and was introduced by Wu [42]

(10) $$u_{sip}=\frac{2}{3}\left(\frac{3-\alpha_{1}l^{2}}{\alpha_{1}}-\frac{3}{2}\frac{1-l^{2}}{K_{n}}\right)\;\beta -\frac{1}{4}\left[l^{4}+\frac{2}{K_{n}^{2}\left(1-l^{2}\right)}\right]\;\left(\beta^{2}\frac{\partial^{2}u}{\partial y^{2}}\right)=A\frac{\partial u}{\partial y}+B\frac{\partial^{2}u}{\partial y^{2}}.$$

In (9), A and B are the constants which infer the slip factors, $l=\min\left[\frac{1}{K_{n}},\;1\right]$, $\alpha_{1}$ symbolizes the momentum accommodation coefficient wherein $0\leq \alpha_{1}\leq 1$, and β as a positive constant represents the molecular mean free path. In addition, with respect to the definition of l, 0≤l≤1 for any value of $K_{n}.$ Further, the first-order velocity slip parameter is denoted by $\gamma =A\sqrt{\frac{a}{\nu_{f}}}$, and γ>0. Meanwhile, the second-order slip parameter is symbolized by $\delta =\frac{Ba}{\nu_{f}}$, and δ<0. From a physical perspective, these slip conditions are applicable in real life flow problems where the slip phenomenon occurs. The most common examples of slip mechanism include fluid motion in the human body, controlling a patient’s blood pressure, micro heat exchangers, interface flows, drug delivery, polishing, and many more. The partial slip conditions are the actual boundary conditions, which occur in real problems.

The modelled flow equations can be translated into a non-dimensional form by utilizing the following similarity parameters (modified from Fang, Yao, Zhang, and Aziz [26]):

(11) $$\eta =y\sqrt{\frac{a}{\nu_{f}}},\;\psi =x\sqrt{a\nu_{f}}f(\eta ),\;\theta \left(\eta \right)=\frac{T-T_{\infty }}{T_{w}-T_{0}}.$$

In above transformation, the Stoke’s stream function ψ(x, y) is defined as $\left(u,\;v\right)=\left(\frac{\partial \psi }{\partial y},-\frac{\partial \psi }{\partial x}\right)$, which satisfies the continuity Equation (1) identically. Now, replacing the similarity variables (11) into Equations (2), (3) and (9) yields the following form of equations:

(12) $$f^{\prime \prime \prime }\left(\eta \right)+\frac{A_{2}}{A_{1}}\left[f\left(\eta \right)\;f^{\prime \prime }\left(\eta \right)-f^{\prime 2}\left(\eta \right)\right]-\frac{K}{A_{1}}f^{\prime }\left(\eta \right)=0,$$

(13) $$\theta^{\prime \prime }\left(\eta \right)+\frac{A_{3}}{A_{4}}\mathrm{Pr}f\left(\eta \right)\;\theta^{\prime }\left(\eta \right)=0,$$

the associated dimensionless boundary conditions become:

(14) $$f(0)=s,\;\;f^{\prime }(0)=\lambda +\gamma f^{\prime \prime }\left(0\right)+\delta f^{\prime \prime \prime }\left(0\right),\;f^{\prime }(\infty )=0,$$

(15) θ(0)=1,  θ(∞)→0,

where the non-dimensional parameters are given as

(16) $$s=-\frac{v_{w}\left(x\right)}{\sqrt{a\nu_{f}}},\;\;\gamma =A\sqrt{\frac{a}{\nu_{f}}},\;\;\delta =B\frac{a}{\nu_{f}},\;\;\mathrm{Pr}=\frac{v_{f}}{\alpha_{f}},\;\;k_{1}=\frac{\rho_{f}ak_{0}}{\mu_{f}}.$$

## 3. Closed-Form Solution for Velocity Field

First, we sought the closed-form solution of the momentum Equation (12) along with the boundary conditions (14). Let us rewrite Equation (12) as:

(17) $$f^{\prime \prime \prime }\left(\eta \right)+A_{5}\left[f\left(\eta \right)\;f^{\prime \prime }\left(\eta \right)-f^{\prime 2}\left(\eta \right)\right]-A_{6}k_{1}f^{\prime }\left(\eta \right)=0,$$

where

(18) A5=A2A1=ρhnfρfμhnfμf and A6=1A1=1μhnfμf.

Suppose that Equation (17) admits the exact solution in the form:

(19) $$f\left(\eta \right)=C_{1}+C_{2}e^{-\beta \eta },$$

Now, making use of Equation (14), the constants $C_{1}$ and $C_{2}$ are written as:

(20) $$C_{1}=s-\frac{\lambda }{\delta \beta^{3}-\gamma \beta^{2}-\beta },$$

(21) $$C_{2}=\frac{\lambda }{\delta \beta^{3}-\gamma \beta^{2}-\beta },$$

Hence, the assumed solution (19) becomes:

(22) $$f\left(\eta \right)=s-\frac{\lambda }{\delta \beta^{3}-\gamma \beta^{2}-\beta }\left(1-e^{-\beta \eta }\right),$$

Next, by using Equation (22) in Equation (17), we find the following algebraic equation:

(23) $$-\frac{\lambda \beta^{2}}{\delta \beta^{2}-\gamma \beta^{2}-1}-A_{5}\left[\frac{\lambda^{2}-s\lambda \beta \left(\delta \beta^{2}-\gamma \beta^{2}-1\right)}{{\left(\delta \beta^{2}-\gamma \beta^{2}-1\right)}^{2}}\right]+A_{6}\frac{\lambda k_{1}}{\delta \beta^{2}-\gamma \beta^{2}-1}=0,$$

Therefore, after simple manipulation, we obtain the following fourth-order algebraic equation in β:

(24) $$\delta \beta^{4}-\left(\gamma +A_{5}\delta s\right)\;\beta^{3}+\left(A_{5}\gamma s-A_{6}k_{1}s-1\right)\;\beta^{2}+\left(A_{5}s+A_{6}\gamma k_{1}\right)\;\beta +\left(A_{5}\lambda +A_{6}k_{1}\right)=0,$$

The most important part of this analysis is the prediction of multiple solutions to the dimensionless problem (17). It should be acknowledged that this question is now turned into finding the positive roots of the polynomial (24), which seem to have a crucial role in determining the dual solutions for the flow field. Moreover, one can see that only positive real and distinct roots present physically feasible solutions. Hence, to obtain the dual solutions, by rewriting Equation (24) in the following form:

(25) $$\beta^{4}-\frac{\left(\gamma +A_{5}\delta s\right)}{\delta }\beta^{3}+\frac{\left(A_{5}\gamma s-A_{6}k_{1}s-1\right)}{\delta }\beta^{2}+\frac{\left(A_{5}s+A_{6}\gamma k_{1}\right)}{\delta }\beta +\frac{\left(A_{5}\lambda +A_{6}k_{1}\right)}{\delta }=0,$$

where it can be written in the alternative form:

(26) $$\beta^{4}-d_{3}\beta^{3}+d_{2}\beta^{2}+d_{1}\beta +d_{0}=0,$$

where:

(27) $$\begin{array}{l} d_{3}=\frac{\left(\gamma +A_{5}\delta s\right)}{\delta },\;\;d_{2}=\frac{\left(A_{5}\gamma s-A_{6}k_{1}s-1\right)}{\delta },\;\\ d_{1}=\frac{\left(A_{5}s+A_{6}\gamma k_{1}\right)}{\delta },\;d_{0}=\frac{\left(A_{5}\lambda +A_{6}k_{1}\right)}{\delta }. \end{array}$$

Introducing a new transformation $z=\beta +\frac{d_{3}}{4},$ we get:

(28) $$z^{4}+Lz^{2}+Mz+N=0,$$

In the above expression, L, $M_{1}$, and N are given as:

(29) $$L=d_{2}-\frac{3}{8}d_{3}^{2},\;M=d_{1}-\frac{1}{2}d_{2}d_{3}+\frac{1}{8}d_{3}^{3},\;N=d_{0}-\frac{1}{4}d_{1}d_{3}+\frac{1}{16}d_{2}d_{3}^{2}-\frac{3}{256}d_{3}^{4}.$$

In particular, the two distinct positive roots of Equation (25) are given by:

(30) $$\beta =\frac{\sqrt{P}}{2}-\frac{1}{2}\sqrt{Q-\frac{2M}{\sqrt{P}}-}\frac{d_{3}}{4},\;\;\beta_{2}=\frac{\sqrt{P}}{2}+\frac{1}{2}\sqrt{Q-\frac{2M}{\sqrt{P}}-}\frac{d_{3}}{4},$$

where

(31) $$\begin{array}{c} P=-\frac{2L}{3}+\frac{2^{\frac{1}{3}}\left(L^{2}+12N\right)}{3{\left(2L^{2}+27M_{1}^{2}-72LN+\sqrt{-4{\left(L^{2}+12N\right)}^{3}+{\left(2L^{2}+27M_{1}^{2}-72LN\right)}^{2}}\right)}^{\frac{1}{3}}}\\ +\frac{3{\left(2L^{2}+27M_{1}^{2}-72LN+\sqrt{-4{\left(L^{2}+12N\right)}^{3}+{\left(2L^{2}+27M_{1}^{2}-72LN\right)}^{2}}\right)}^{\frac{1}{3}}}{3\times 2^{\frac{1}{3}}}, \end{array}$$

(32) $$\begin{array}{c} P=-\frac{4L}{3}-\frac{2^{\frac{1}{3}}\left(L^{2}+12N\right)}{3{\left(2L^{2}+27M_{1}^{2}-72LN+\sqrt{-4{\left(L^{2}+12N\right)}^{3}+{\left(2L^{2}+27M_{1}^{2}-72LN\right)}^{2}}\right)}^{\frac{1}{3}}}\\ -\frac{3{\left(2L^{2}+27M_{1}^{2}-72LN+\sqrt{-4{\left(L^{2}+12N\right)}^{3}+{\left(2L^{2}+27M_{1}^{2}-72LN\right)}^{2}}\right)}^{\frac{1}{3}}}{3\times 2^{\frac{1}{3}}}. \end{array}$$

The velocity field is determined for both a stretching or shrinking sheet after differentiating Equation (22) as:

(33) $$f^{\prime }\left(\eta \right)=\frac{\lambda }{\left(1+\gamma \beta -\delta \beta^{2}\right)}e^{-\beta \eta }.$$

## 4. Skin-Friction Coefficient

The skin-friction coefficient $C_{f}$ is expressed as:

(34) $$C_{f}=\frac{\tau_{w}}{\rho_{f}U_{w}^{2}},$$

where $\tau_{w}$ is given by:

(35) $$\tau_{w}={\mu_{nf}\left(\frac{\partial u}{\partial y}\right)|}_{y=0},$$

Upon using Equations (11) and (35), the skin-friction coefficient is calculated with the next formula:

(36) (Re)1/2Cf=1A1f″(0)=1A1βλδβ2−γβ−1,

where Re=xUw/νf represents the Reynolds number.

## 5. Numerical Solution for Temperature Field

The numerical solution for the temperature field θ(η) was computed by implementing the bvp4c routine in MATLAB. For this purpose, first we invoked the obtained closed-form solution for the dimensionless stream function f(η) from Equation (22) into Equation (13). Then, the resulting ODE (13) with (15) was tackled numerically via the bvp4c routine. For this, let $\theta =Y_{1}$ and $\theta^{\prime }=Y_{2}$, such that

(37) $$\begin{array}{l} \theta_{1}^{\prime }=\theta_{2},\;\\ \theta_{2}^{\prime }=-\frac{A_{3}}{A_{4}}\mathrm{Pr}fY_{2}, \end{array}$$

and the associated boundary conditions are

(38) $$Y_{1}\left(0\right)=1,\;\;Y_{1}\left(\infty \right)\to 0.$$

## 6. Physical Outcomes and Discussion

To describe the physical outcomes of current problem, the graphical results are presented in terms of the flow and temperature fields for the various physical parameters. We investigated the fluid flow and heat transport attributes by computing the profiles of non-dimensional velocity and temperature for the water-based hybrid nanofluid mixed with $Al_{2}O_{3}/SiO_{2}$ nanoparticles. In all the profiles, one can see the dual solutions (upper and lower) in the case of shrinking-sheet flow (λ<0) for a limited range of the suction parameter. This analysis was performed by varying the involved physical parameters such as the stretching/shrinking parameter, mass suction parameter, porosity parameter, nanoparticle volume fractions, and the first- and second-order slip parameters. All these flow parameters have a significant role in various fields of engineering, biomedicine, and the chemical industry as well as other fields of science. Meanwhile, the following fixed values for the involved physical parameters were used within this analysis: λ=−1.5, δ=−1, γ=1=K=s, $M=0.5,\;\varphi_{1}=0.1=\varphi_{2},\;\mathrm{Pr}=6.2$.

### 6.1. Validation

To check the validity of the computed results, the present results of the solution values β and the skin-friction coefficient ReCf were compared with the results of Turkyilmazoglu [27] in Table 2 and Table 3. Both the tables reveal that the computed results were very accurate.

### 6.2. Solution Domain

**Figure 2:**
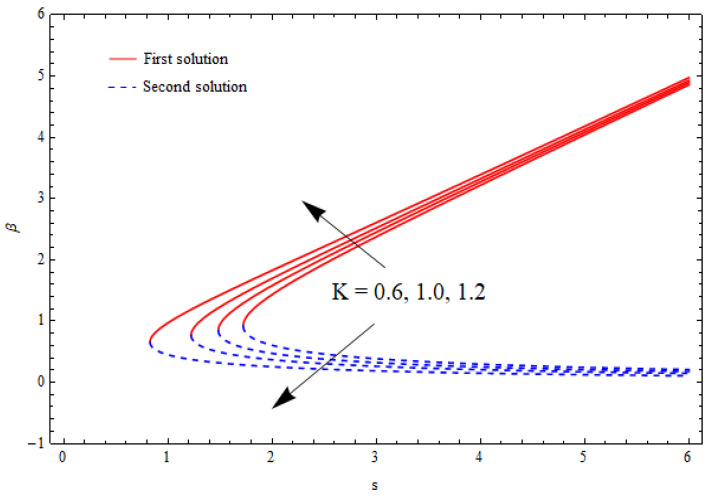
The solution domain β against mass suction parameter s for distinct $k_{1}$ when $\lambda =-1.5,\;\gamma =1,\;\delta =-1,\;\varphi_{1}=0.1=\varphi_{2}$.

**Figure 3:**
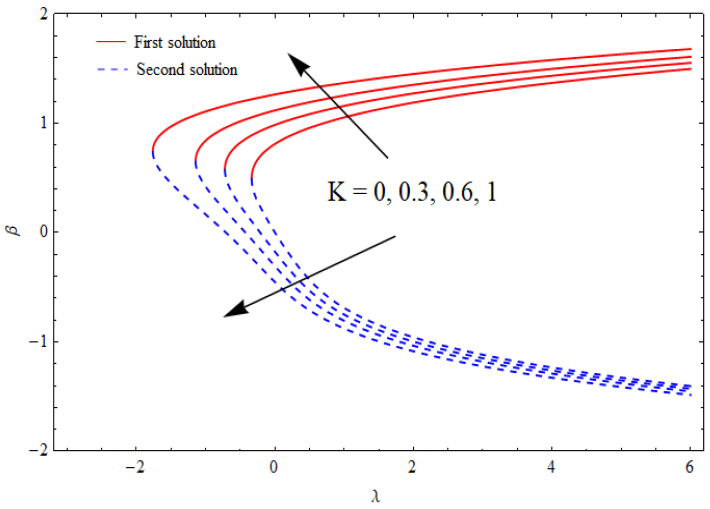
The solution domain β against stretching/shrinking parameter λ for distinct $k_{1}$ when $s=1=\gamma ,\;\delta =-1,\;\varphi_{1}=0.1=\varphi_{2}$.

**Figure 4:**
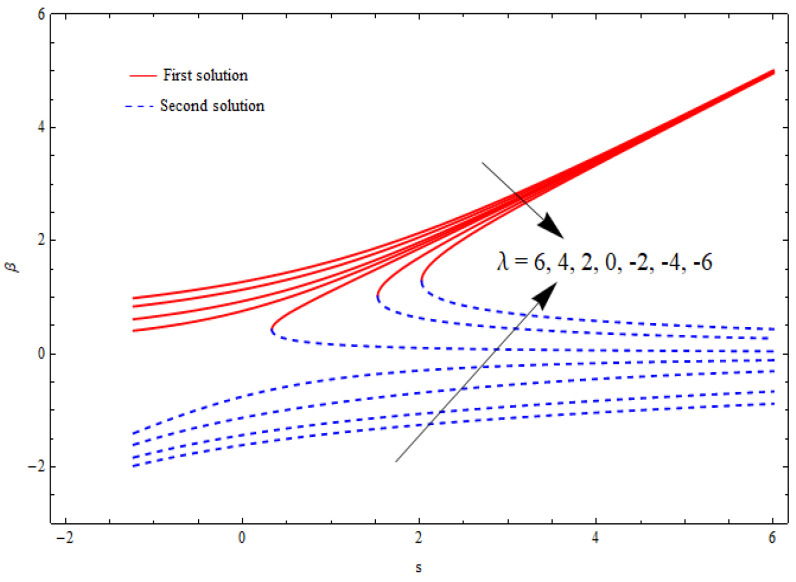
The solution domain β against mass suction parameter s for distinct λ when $k_{1}=1=\gamma ,\;\delta =-1,\;\varphi_{1}=0.1=\varphi_{2}.$

**Figure 5:**
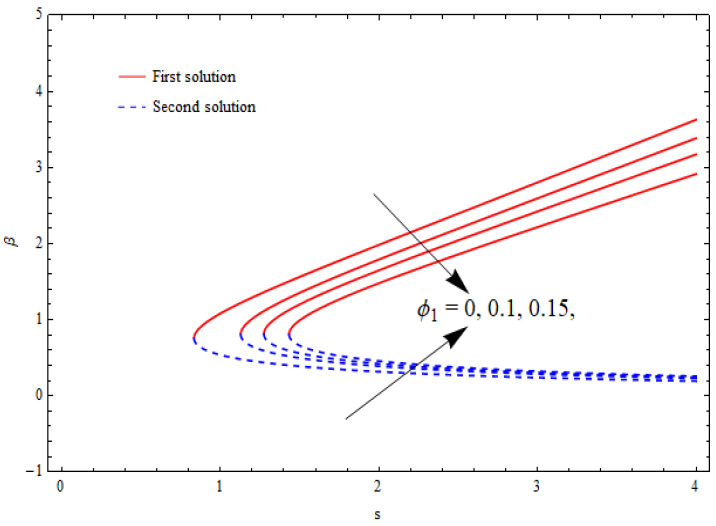
The solution domain β against mass suction parameter s for distinct $\varphi_{1}$ when $\lambda =-1.5,\;k_{1}=1=\gamma ,\;\delta =-1,\;\varphi_{2}=0.1.$

Figure 2, Figure 3, Figure 4 and Figure 5 show the relationship between the solution domain β and the stretching/shrinking parameter (λ), porosity parameter $\left(k_{1}\right),$ nanoparticle volume fractions $\left(\varphi_{1},\;\varphi_{2}\right)$, and the suction parameter (s) for both the upper- and lower-branch solutions. From these figures, it was noted that the solution profiles were significantly affected by the porosity parameter $\left(k_{1}\right)$ as a function of the suction and shrinking parameters. It can be seen from Figure 2 and Figure 3 that an increase in $k_{1}$ gave a gradual increment to the first solutions of β and reduced the second solutions of β. Furthermore, the solution domain β became wider with the uplifting values of the porosity parameter. Apparently, Figure 4 reveals that the solution profiles β were enhanced with a higher value of the stretching/shrinking parameter λ for both solutions. In Figure 5, the solution domain is captured against the mass suction parameter with distinct values of the nanoparticle volume fraction $\varphi_{1}$. As expected, the solution profiles decreased for the first solution and were enhanced in the second solution with the growing nanoparticle volume fraction $\varphi_{1}$.

### 6.3. Flow Analysis

**Figure 6:**
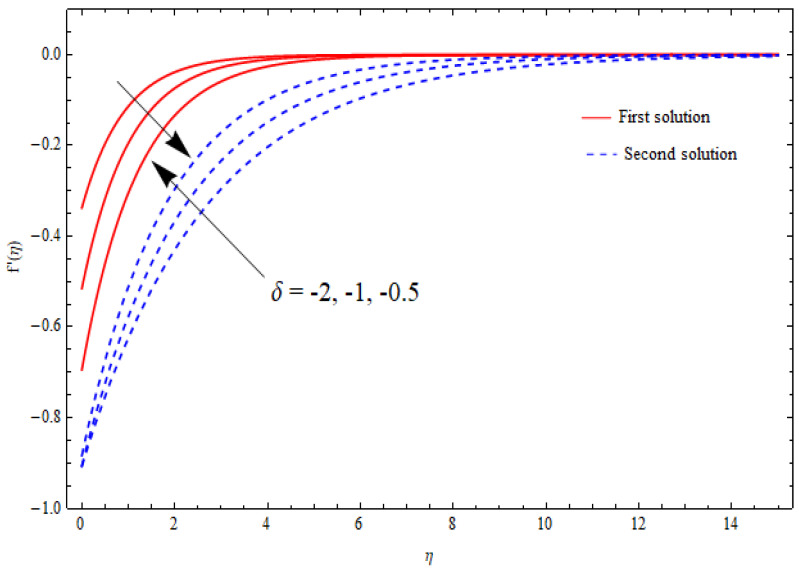
Velocity profiles $f^{\prime }\left(\eta \right)$ for distinct δ when $\lambda =-1.5,\;k_{1}=1=\gamma =s,\;\varphi_{1}=0.1=\varphi_{2}.$

**Figure 7:**
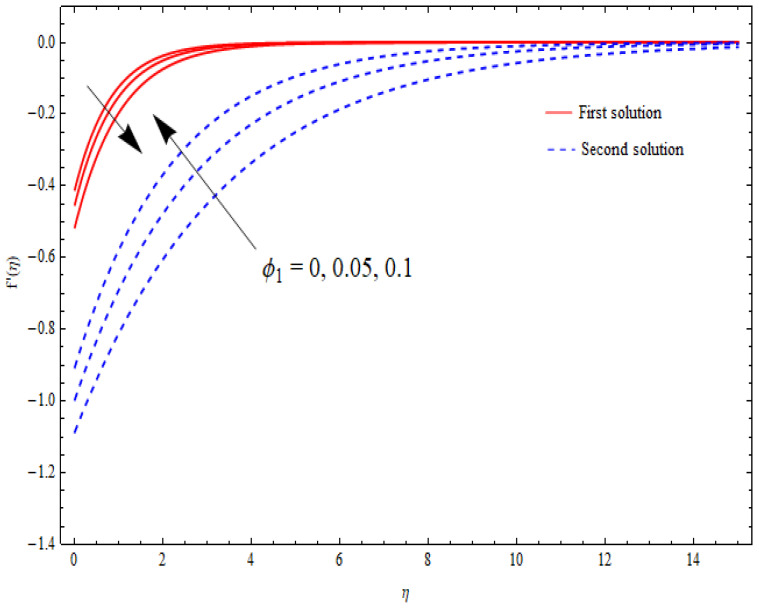
Velocity profiles $f^{\prime }\left(\eta \right)$ for distinct $\varphi_{1}$ when $\lambda =-1.5,\;s=K=\gamma =1,\;\delta =-1,\;\phi_{2}=0.1.$

**Figure 8:**
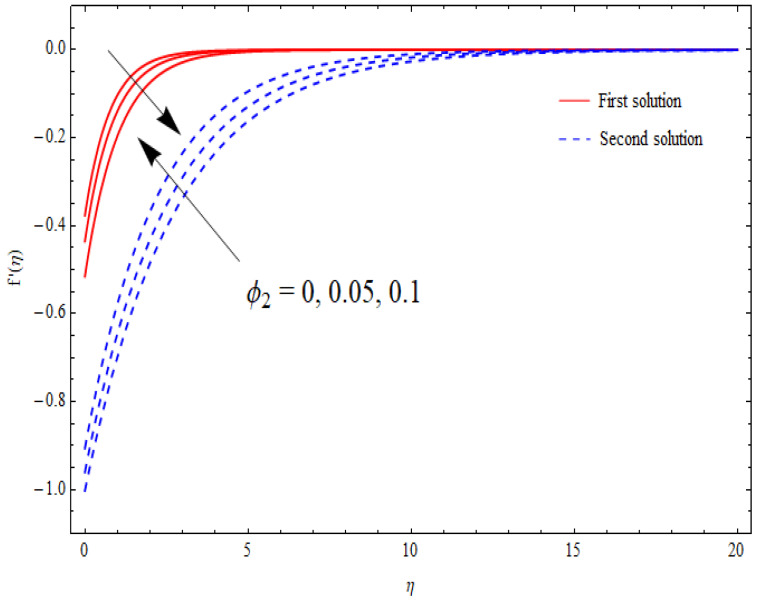
Velocity profiles $f^{\prime }\left(\eta \right)$ for distinct $\varphi_{2}$ when $\lambda =-1.5,\;k_{1}=1=\gamma =s,\;\delta =-1,\;\varphi_{1}=0.1.$

**Figure 9:**
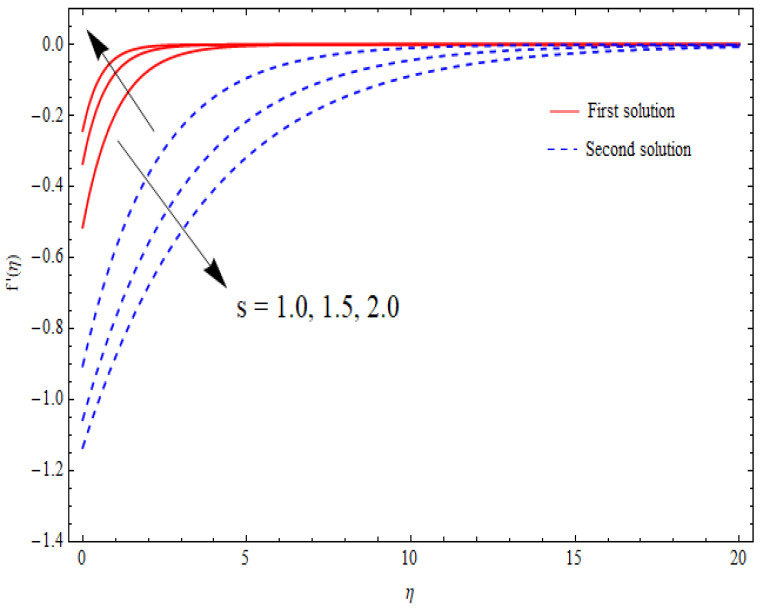
Velocity profiles $f^{\prime }\left(\eta \right)$ for distinct s when $\lambda =-1.5,\;k_{1}=1=\gamma ,\;\delta =-1,\;\varphi_{1}=0.1=\varphi_{2}.$

**Figure 10:**
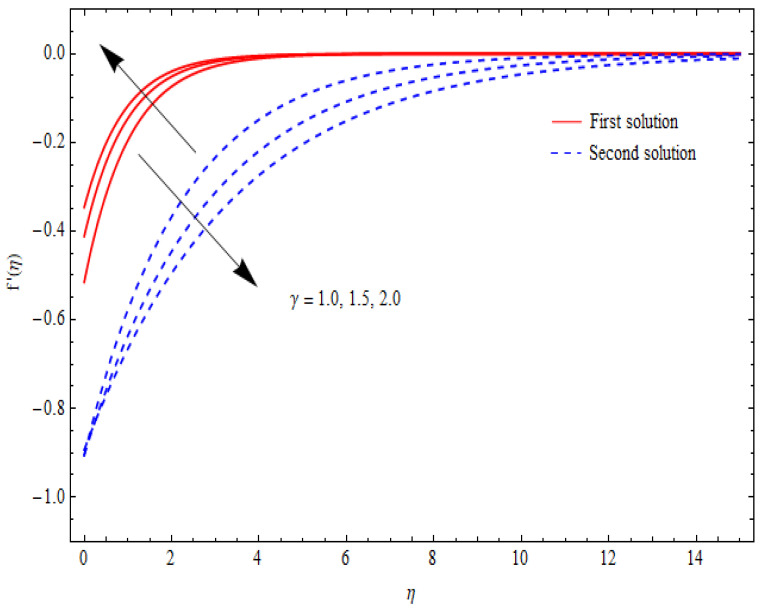
Velocity profiles $f^{\prime }\left(\eta \right)$ for distinct γ when $\lambda =-1.5,\;k_{1}=1=s,\;\delta =-1,\;\varphi_{1}=0.1=\varphi_{2}.$

**Figure 11:**
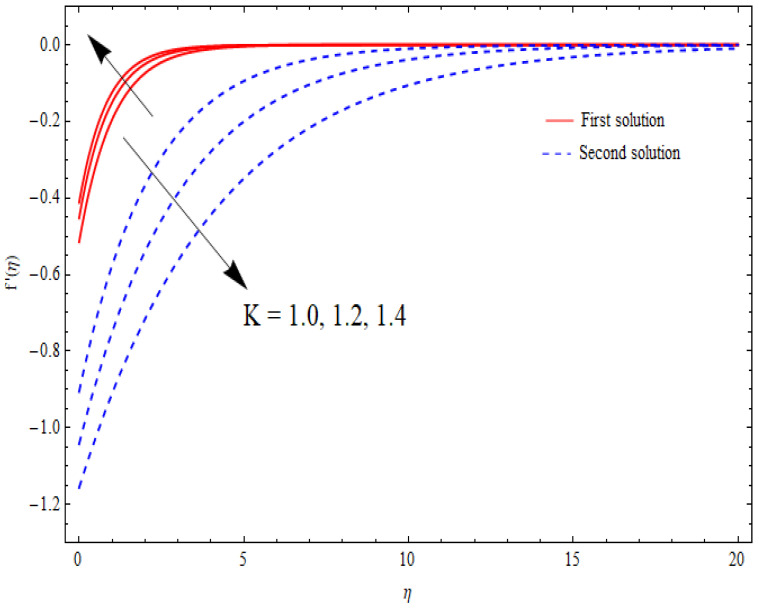
Velocity profiles $f^{\prime }\left(\eta \right)$ for distinct $k_{1}$ when $\lambda =-1.5,\;s=1=\gamma ,\;\delta =-1,\;\varphi_{1}=0.1=\varphi_{2}.$

**Figure 12:**
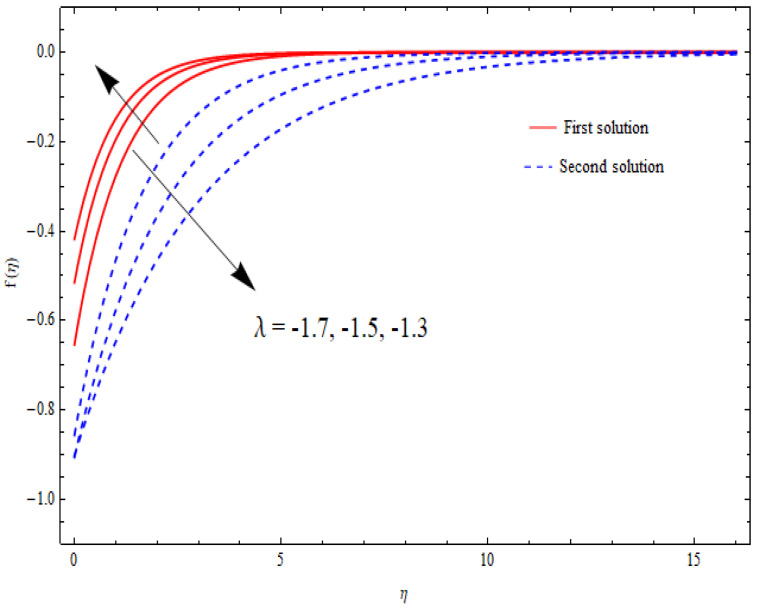
Velocity profiles $f^{\prime }\left(\eta \right)$ for distinct λ when $s=k_{1}=1=\gamma ,\;\delta =-1,\;\varphi_{1}=0.1=\varphi_{2}.$

The variations in the dual velocity curves $f^{\prime }\left(\eta \right)$ for varying values of the second-order slip parameter (δ), nanoparticle volume fractions $\left(\varphi_{1},\;\varphi_{2}\right),$ mass suction parameter (s), first-order slip parameter (γ), porosity parameter $\left(k_{1}\right)$, and stretching/shrinking parameter (λ) inside the boundary layer regime are displayed through Figure 6, Figure 7, Figure 8, Figure 9, Figure 10, Figure 11 and Figure 12, respectively. It is worth noting that all the graphs display the existence of dual solutions for shrinking flow and satisfied the far-field boundary conditions asymptotically. Here, the velocity curves in the case of the first solution were decremented with a higher value of δ, $\varphi_{1}$, and $\varphi_{2}$, while the opposite was seen for the second solution, as seen in Figure 6, Figure 7 and Figure 8, respectively. The profile of velocity for the $Al_{2}O_{3}/SiO_{2}$ hybrid nanofluid is sketched in Figure 9 and Figure 10 due to the variation in the suction and the first-order slip parameter, respectively. It can be seen through these figures that the velocity distribution was enhanced by increasing s for the upper-branch solution and reduce for the lower-branch solution. Moreover, similar behavior was noted for the corresponding momentum boundary layer. It should also be observed that the velocity graph increased with higher values of the first-order slip parameter γ in the upper-branch solution, but in the lower-branch solution it showed a decreasing trend. The effect of the porosity parameter $k_{1}$ on the non-dimensional velocity distributions $f^{\prime }\left(\eta \right)$ is shown in Figure 11. The sketched velocity profiles demonstrate the increasing behavior with the increased value of $k_{1}$, in the case of the upper-branch solution, while decreasing behavior was perceived for the lower-branch solution. Figure 12 is plotted showing the relationship between the stretching/shrinking parameter λ and the velocity field $f^{\prime }\left(\eta \right)$ by keeping other parameters’ values fixed. According to Figure 12, the velocity curves increased with a higher value of λ in the upper-branch solution, while it decreased in the lower-branch solutions.

### 6.4. Skin-Friction Coefficient

**Figure 13:**
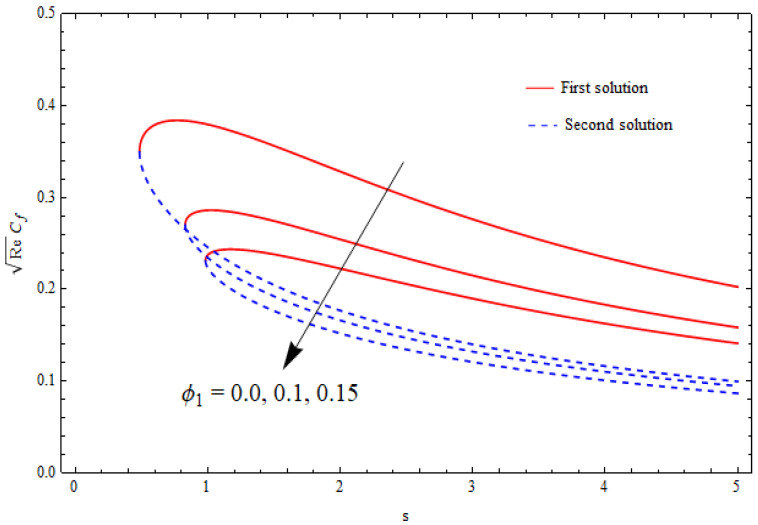
Variation in the skin-friction coefficient ReCf against s for different $\varphi_{1}$ when $\lambda =-1.5,\;k_{1}=1=\gamma ,\;\delta =-1,\;\varphi_{2}=0.1.$

**Figure 14:**
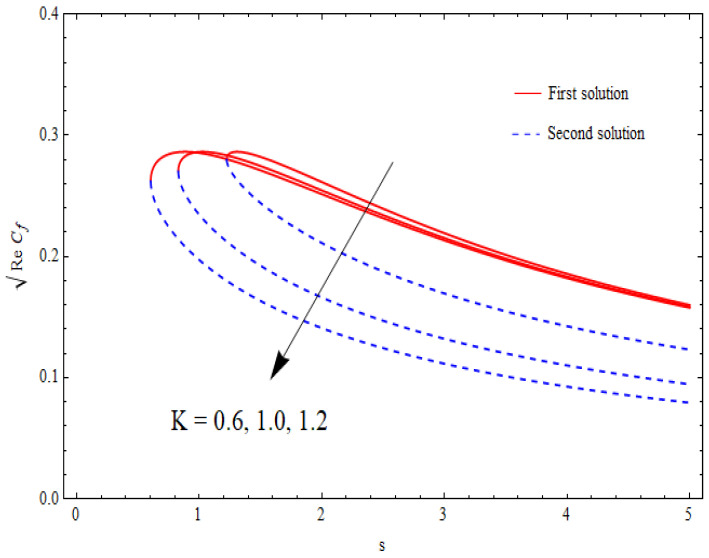
Variation in the skin-friction coefficient ReCf against s for different $k_{1}$ when $\lambda =-1.5,\;\gamma =1,\;\delta =-1,\;\varphi_{1}=0.1=\varphi_{2}.$

The graphical data for the skin-friction coefficient ReCf against the suction parameter s for distinct values of $\varphi_{1}$ and $k_{1}$ are demonstrated in Figure 13 and Figure 14. Again, this data suggested the existence of dual solutions for a specific range of mass suction past a shrinking sheet. It was further depicted from these figures that the critical values of mass suction $\left(s_{critical}\right)$ lay in the range $0<s_{critical}<1$ for $\varphi_{1}=0.0,\;0.05,\;0.1$ and $k_{1}=1.0,\;1.2,\;1.6$. In addition, the critical values in Figure 13 and Figure 14 decreased with higher values of these parameters. The role of the nanoparticle volume fraction was to significantly enhance the skin-friction coefficient for both solutions. Contrary results were seen for the porosity parameter $k_{1}$.

### 6.5. Thermal Analysis

**Figure 15:**
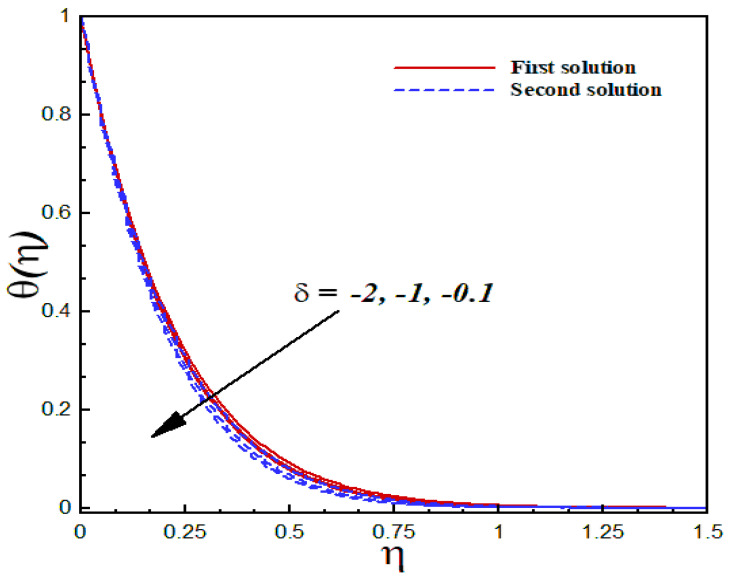
Temperature profiles θ(η) for distinct δ when $\lambda =-1.9,\;k_{1}=1=\gamma =s,\;\varphi_{1}=0.1=\varphi_{2},\;\mathrm{Pr}=6.2.$

**Figure 16:**
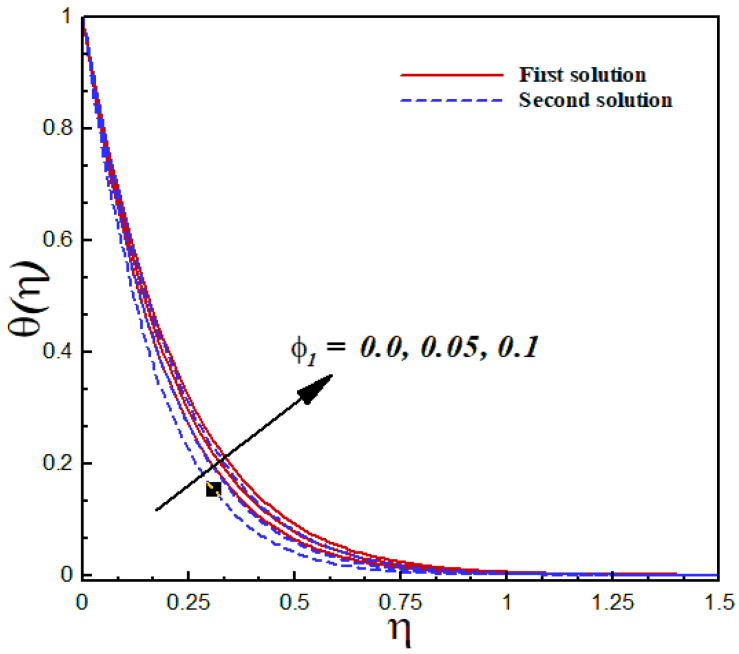
Temperature profiles θ(η) for distinct $\varphi_{1}$ when $\lambda =-1.9,\;k_{1}=1=\gamma =s,\;\delta =-2,\;\varphi_{2}=0.1,\;\mathrm{Pr}=6.2.$

**Figure 17:**
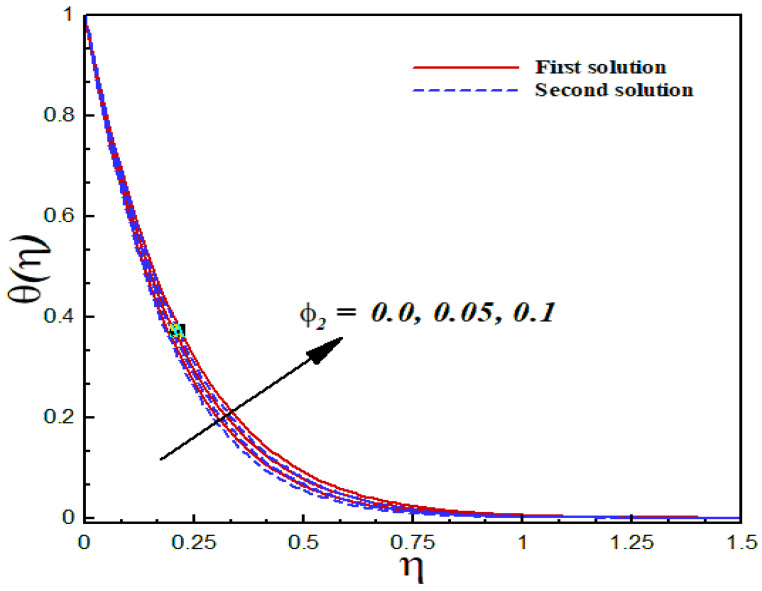
Temperature profiles θ(η) for distinct $\varphi_{2}$ when $\lambda =-1.5,\;k_{1}=1=\gamma =s,\;\delta =-2,\;\varphi_{1}=0.1,\;\mathrm{Pr}=6.2.$

**Figure 18:**
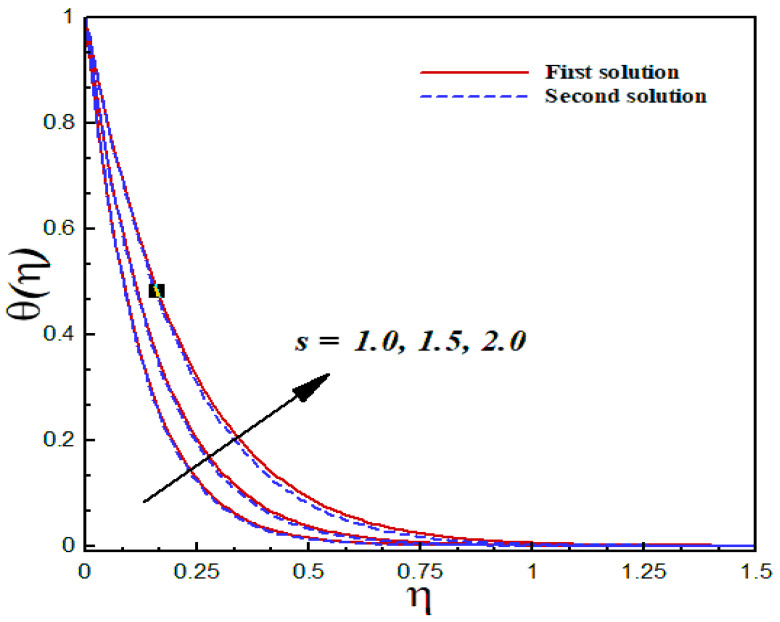
Temperature profiles θ(η) for distinct s when $\lambda =-1.5,\;k_{1}=1=\gamma ,\;\delta =-2,\;\varphi_{1}=0.1=\varphi_{2},\;\mathrm{Pr}=6.2.$

The thermal characteristics of the $Al_{2}O_{3}/SiO_{2}$ hybrid nanofluid flow are illustrated graphically through Figure 15, Figure 16, Figure 17 and Figure 18 for several values of the physical parameters δ, $\varphi_{1},$ $\varphi_{2}$, and s. In each figure, we observed the dual nature of the temperature profiles θ(η) for shrinking flow with specific mass suction. The effects of the second-order slip parameter δ on the dimensionless temperature profiles inside the boundary layer are displayed in Figure 15. As expected, the temperature curves showed a decreasing behavior with an uplifting value of the second-order slip parameter in both solutions. The computed results of the thermal transport of the $Al_{2}O_{3}/SiO_{2}$ hybrid nanofluid are illustrated in Figure 16 and Figure 17 in the form of temperature fields with several values of the nanoparticle volume fraction. According to these figures, an increasing value of the nanoparticle volume fraction significantly enhanced the temperature field for both the solutions. Figure 18 reveals the outcome of the suction parameter s on the dimensionless temperature distributions θ(η) inside the boundary layer region. The suction parameter significantly affected the temperature curves and showed an increasing trend with a greater value of s.

## 7. Conclusions

In this research work, we investigated the flow of a water-based ${Al}_{2}O_{3}/SiO_{2}$ hybrid nanofluid against a permeable stretching/shrinking sheet within a porous medium in the presence of partial slip conditions. The governing system of highly non-linear differential equations was solved both analytically and numerically for the velocity and temperature fields. The most significant observations of the current analysis are as follows:

- The velocity distributions displayed different behaviors for the increasing porosity parameter.
- Dual solutions (upper-branch solution and lower-branch solution) existed for shrinking flow within a specific range of the suction parameter.
- A significant reduction in friction at the wall was noticed for a higher porosity parameter.
- The solution range was significantly increased with increasing values of the porosity parameter.
- A decrement in the velocity profiles for upper branch solution was noted against the nanoparticle volume fraction.
- The skin-friction coefficient showed a substantial enhancement with a higher nanoparticle volume fraction for both the solutions.

## Figures and Tables

**Table 1:** Thermo-physical properties of the base fluid and different nanoparticles.

Physical Properties	Base Liquid	Nanoparticles	
	$H_{2}O$	$Al_{2}O_{3}$	$SiO_{2}$
$\rho \left(kg/m^{3}\right)$	997.1	3970	2200
$c_{p}\left(J/kgK\right)$	4179	765	703
k(W/mK)	0.613	40	1.2
σ(S/m)	$5.5\times 10^{-6}$	$35\times 10^{6}$	$10^{-23}$

**Table 2:** Comparison of numerical values of solution β as a function of s and γ when $\varphi_{1}=0=\varphi_{2}=k_{1},\;\lambda =-1=\delta .$

*s*	*γ*	Upper Branch		Lower Branch	
		Turkyilmazoglu [27]	Present Study	Turkyilmazoglu [27]	Present Study
2	0	1.8832035	1.883203505	0.53101006	0.531010056
-	1	1.9212896	1.921289609	0.40052899	0.400528985
-	3	1.9519690	1.951968975	0.29676823	0.296768226
-	10	1.9795599	1.979559862	0.18882968	0.188829678
3	0	2.9655726	2.965572633	0.33720300	0.337203003
-	1	2.9737635	2.9737634890	0.27228263	0.272282625
-	3	2.9822017	2.982201678	0.21301670	0.213016696
-	10	2.9916152	2.991615203	0.14289554	0.142895541
5	0	4.9922728	4.992272838	0.20030956	0.200309564
-	1	4.9935252	4.993525154	0.17232557	0.172325571
-	3	4.9951096	4.995109595	0.14226228	0.142262284
-	10	4.9973652	4.997365206	0.10102758	0.101027578

**Table 3:** Comparison values of $-f^{\prime \prime }\left(0\right)$ with existing works when $\varphi_{1}=0=\varphi_{2}=k_{1},$ λ=1, s=2.

*γ*	*δ*	Turkyilmazoglu [27]	Present Study
0	0	2.41421356	2.414213547
-	−1	0.38942826	0.389428256
-	−3	0.15150097	0.151500971
-	−5	0.09425550	0.094255503
1	0	0.68232780	0.682327801
-	−1	0.28168830	0.281688300
-	−3	0.13178867	0.131788669
-	−5	0.08620469	0.086204692
3	0	0.28704681	0.287046812
-	−1	0.18074365	0.180743647
-	−3	0.10449187	0.104491866
-	−5	0.07360578	0.073605784

## Data Availability

Not applicable.
